# Geographic distribution of the Birmingham Darter *Etheostoma birminghamense*

**DOI:** 10.1101/2025.11.05.686853

**Published:** 2025-11-07

**Authors:** Chase D. Brownstein, Konstantinos Andriotis, Christopher B. Haynes, Nathaniel D. Sturm, Bernard R. Kuhajda, Thomas J. Near

**Affiliations:** 1 Department of Ecology and Evolutionary Biology, Yale University, New Haven CT, USA; 2 Geological Survey of Alabama, Tuscaloosa AL, USA; 3 Tennessee Aquarium Conservation Institute, Chattanooga TN, USA; 4 Yale Peabody Museum, New Haven CT, USA

**Keywords:** Microendemism, Darter, Range Extension, Carbonate, Alabama, Black Warrior River

## Abstract

Southeastern North America harbors the richest freshwater biodiversity hotspot in the northern hemisphere and is home to numerous species with extremely narrow ranges. Among these are the six species of the *Etheostoma chermocki* species complex, which exclusively inhabit small streams spanning fewer than 100 square kilometers in Alabama, USA. One of these species, the Birmingham Darter *Etheostoma birminghamense*, was described in April 2025 from Valley Creek and its associated tributaries, which extend into the urban core of Birmingham, AL, and its suburbs. At least one population of *E. birminghamense* is feared extirpated, highlighting the imperilment of this microendemic species. Here, we report the results of recent collections that extend the range extension of *E. birminghamense* into Little Blue Creek, Nabors Branch, Halls Creek, and localities in the mainstem of Valley Creek. As previously hypothesized, occurrences of *E. birminghamense* are associated with exhumed Cambrian-Ordovician- and Mississippian-age carbonate units, which in Little Blue Creek appear only as pockets of exposed bedrock at the base of the channel and on the banks. Our observations demonstrate that *E. birminghamense* is distributed throughout the majority of the Valley Creek drainage, highlighting the need for rapid assessment of its conservation status.

## Introduction.

Darters (*Percidae*: *Etheostomatinae*) are one of the most species-rich lineages of freshwater vertebrates inhabiting the southeastern North American freshwater biodiversity hotspot (Near et al. 2011; Page 1983). The diversity of this clade, which is endemic to North America, is asymmetrically distributed across this hotspot. The springs and creeks around the city of Birmingham in the US state of Alabama harbor a number of endemic species with very small ranges. These include the Federally Listed Rush Darter *Etheostoma phytophilum* Bart and Taylor 1999, Watercress Darter *Etheostoma nuchale* Howell and Caldwell 1965, and Vermillion Darter *Etheostoma chermocki* Boschung, Mayden and Tomelleri 1992. The Vermillion Darter is one of six species that comprise the *Etheostoma chermocki* species complex (Boschung et al. 1992; Clabaugh et al. 1996; Mayden and Kuhajda 2025; Brownstein et al. 2025; Kim et al. 2023).

Four of the six species of the *Etheostoma chermocki* species complex were recently delimited and described on the basis of a combination of genomic data and meristic and coloration traits (Brownstein et al. 2025; Kim et al. 2023; Mayden and Kuhajda 2025). These species, which last shared common ancestry more than one million years ago, face threats from stream degradation due to urbanization along with the Rush and Watercress Darters (Brownstein et al. 2025; Khudamrongsawat and Kuhajda 2007; Khudamrongsawat et al. 2005; Duncan et al. 2010; 2016; Fluker et al. 2010; 2009; Howell et al. 2016). The small ranges of these microendemic darter species and their proximity to urban centers makes updated and detailed documentation of their distribution essential (Howell et al. 2016; Brownstein et al. 2025). In the case of the *Etheostoma chermocki* complex, the close association of these species with carbonate bedrocks, which crop out only at specific sections of streams they occupy (Brownstein et al. 2025; Kim et al. 2023), suggests that even small habitat changes driven by anthropogenic activity could extirpate these ancient lineages or drive species to extinction.

Here, we report range extensions for the Birmingham Darter *Etheostoma birminghamense* Brownstein, Kim, Wood, Alley, Stokes, and Near 2025, which is endemic to the Valley Creek system around Birmingham and the adjacent city of Bessemer, Alabama (Brownstein et al. 2025). The Birmingham Darter population from Fivemile Creek has not been observed since 2006, underscoring its imperilment (Brownstein et al. 2025). We document the occurrence of the Birmingham Darter in Little Blue Creek, Halls Creek, and Nabors Branch, which fall within the lower, middle, and upper portions of the upper Valley Creek system. Although carbonate bedrock is not discernable from geological maps of some of the collection sites (Little Blue Creek), we documented its presence as these small streams erode through the capping layer of siliciclastic rock.

## Results and Discussion.

**Figure 1.**
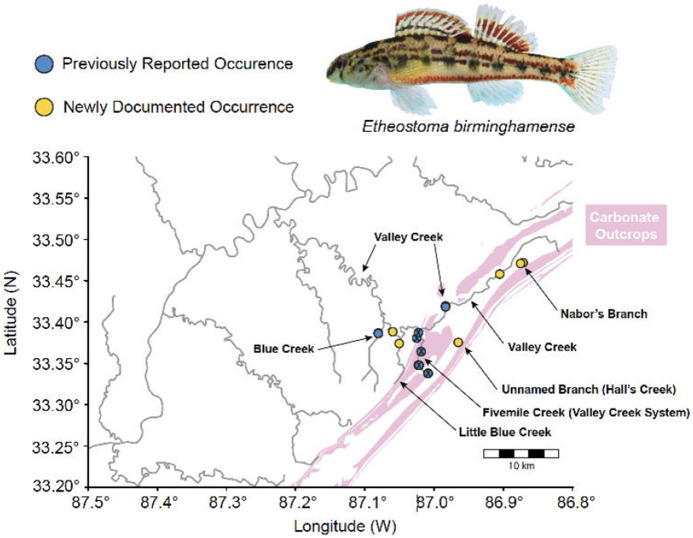
Locality map of Birmingham Darter *Etheostoma birminghamense*. Map shows rivers and streams in the Black Warrior River system and carbonate outcrops associated with them. Dots indicate localities from which *E. birminghamense* has been sampled. X marks on locality dots indicate extirpated populations. Photograph of Birmingham Darter by Julia E. Wood and Zachariah D. Alley, used with permission.

We report *Etheostoma birminghamense* from six new localities all in Jefferson Co., Alabama: Little Blue Creek at CDX Gas Road crossing approximately 1.75 km southeast of Johns Road (AL 36), Bessemer; Valley Creek at shoal approximately 200 m downstream of Johns Road (AL 36) crossing, Bessemer; Valley Creek at B.Y. Williams Sr. Drive, Birmingham; unnamed tributary to Halls Creek near the Watercress Darter National Wildlife Refuge (NWR), approximately 60 m downstream of the culvert at the intersection of South Division Street and Division Court, Bessemer; Nabors Branch near 24th St SW, Birmingham, and in Seven Springs run (Nabors Branch tributary) along Cleburne Avenue, Birmingham (Figure 1).

Biologists from Geological Survey of Alabama (including CBH, NDS) conducted a fish survey in an unnamed tributary to Halls Creek near the Watercress Darter NWR on December 4, 2024, during which three *Etheostoma birminghamense* [No voucher] were captured via seining approximately 60 m downstream of the culvert located at the intersection of South Division Street and Division Court. The three darters were originally identified as members of the *Etheostoma bellator* Suttkus and Bailey 1993, as *E. birminghamense* had not been formally described at the time. At the time of the survey, the current was slow, and the stream bed was primarily covered in coarse organic matter and detritus, with patches of gravel and exposed carbonate bedrock. Species that co-occur with *Etheostoma birminghamense* in the unnamed tributary to Halls Creek include Creek Chub *Semotilus atromaculatus*, Largescale Stoneroller *Campostoma oligolepis*, Blackspotted Topminnow *Fundulus olivaceus*, Western Mosquitofish *Gambusia affinis*, Bluegill *Lepomis macrochirus*, Gulf Longear Sunfish *Lepomis solis,* Alabama Bass *Micropterus henshalli,* Warrior Bass *Micropterus warriorensis,* Redspot Darter *Etheostoma artesiae*, Speckled Darter *Etheostoma stigmaeum*, and Watercress Darter *Etheostoma nuchale*.

**Figure 2.**
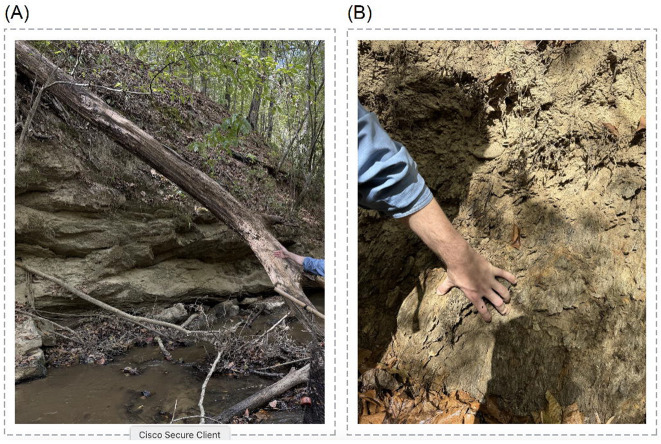
Examples of bedrock outcrops along Little Blue Creek. Photographs by KA, hand of CDB for scale. (A) Outcrops of overlying soil and siliciclastic rock on bank of Little Blue Creek, and (B) closeup of non-carbonate rock on side of stream.

Three of us (CDB, KA, TJN) collected three specimens [Yale Peabody Museum Ichthyology Collections (YPM ICH) XXXXX] of *Etheostoma birminghamense* from Little Blue Creek on October 11, 2025. We caught these specimens by seining along isolated patches of carbonate bedrock with moderate current on the stream margins. At this location, carbonate is barely exposed along the stream bed, which gravel and silt cover at its middle, while siliciclastic cap rock and soil emarginate its banks (Figure 2). Carbonate does not appear on geological maps of Cambrian-Ordovician and Mississippian outcrops of this stream (Figure 1) and is restricted to the margins of the stream bed.

Species that co-occur with *Etheostoma birminghamense* in Little Blue Creek include Blacktail Shiner *Cyprinella venusta*, Creek Chub *Semotilus atromaculatus*, *Campostoma oligolepis*, *Gambusia affinis*, *Lepomis macrochirus*, *Etheostoma artesiae*, and Blackbanded Darter *Percina nigrofasciata*. Upstream of this locality in Little Blue Creek off Mt. Forest Road, we collected numerous individuals of *E. artesiae*, as well as *Semotilus atromaculatus*, *Gambusia affinis*, and Gulf Longear Sunfish *Lepomis solis*, but did not observe the presence of *E. birminghamense*. Habitat at this upstream locality consisted of small, sporadically connected pools in the dry channel of Little Blue Creek.

In comparison, *Etheostoma birminghamense* is far more abundant in the main stem of Valley Creek approximately 200 m from Johns Road, Bessemer (18 individuals; YPM ICH XXXXX) and at B. Y. Williams Sr. Drive in Birmingham (64 individuals). At the Johns Road Valley Creek locality, *E. birminghamense* was abundant even though carbonate is not apparent on outcrop maps at this locality but was clearly present in the stream where it has been exhumed. We collected a small specimen of carbonate rock from this locality and is associated with the *E. birminghamense* specimen lot from this site in the YPM ichthyology collection. *Etheostoma birminghamense* appears especially abundant on carbonate that has been colonized by invertebrates and aquatic vegetation compared to uncolonized, slippery platforms. Here, co-occurring species include Alabama Hogsucker *Hypentelium etowanum* and *Percina nigrofasciata*.

On January 31, 2020, one of us (BRK) and colleagues collected over 40 *Etheostoma birminghamense* in Nabors Branch both above and below the mouth of Seven Springs run over gravel, cobble, and bedrock in slow to moderate flow. Other fishes present included *Campostoma oligolepis*, *Cyprinella venusta*, Silverstripe Shiner *Notropis stilbius*, *Semotilus atromaculatus*, *Hypentelium etowanum*, *Gambusia affinis*, *Lepomis solis, E. nuchale*, *E. stigmaeum*, and *Percina nigrofasciata*. This site was revisited on October 17, 2025 and 4 *E. birminghamense* were collected along with Banded Sculpin *Cottus carolinae*. Nabors Branch is the uppermost stream harboring *E. birminghamense* in the Valley Creek watershed and is a second stronghold for the species. Seven Springs is a small tributary to Nabors Branch, and on each of two dates (March 3, 2018 and October 17, 2025) a single *E. birminghamense* was collected over a silt and small gravel bottom with slow flow. Other fishes present included *Campostoma oligolepis*, Striped Shiner *Luxilus chrysocephalus*, *Semotilus atromaculatus*, *Hypentelium etowanum*, *Gambusia affinis*, and Green Sunfish *Lepomis cyanellus*.

On October 12, 2025, three of us (CDB, KA, TJN) captured only one individual of *Etheostoma birminghamense* (YPM ICH XXXXX) from the type locality of the species at Blue Creek, which was characterized by very low flow and moderate sedimentation. In Blue Creek, *E. birminghamense* co-occurs with *Hypentelium etowanum*, *Campostoma oligolepis*, Warrior Shiner *Lythrurus alegnotus*, Burrhead Shiner *Alburnops asperifrons*, *Notropis stilbius*, Speckled Darter *Etheostoma stigmaeum*, and *Percina nigrofasciata.*

The expansion of the known geographic distribution of the Birmingham Darter *Etheostoma birminghamense* reported in this manuscript suggests the species is widely distributed in the Valley Creek system in regions where there is sufficient water flow and outcropping carbonates. This supports the hypothesis that members of the *Etheostoma chermocki* species complex have speciated in a manner mediated by dispersal to areas in the upper Black Warrior River system with exposed carbonate bedrock (Kim et al. 2023). As previously noted, all members of this species complex face major risks to their survival from anthropogenic activity (Mayden and Kuhajda 2025; Brownstein et al. 2025). Specific threats to the Valley Creek watershed and *E. birminghamense* include an urban landscape (Brownstein et al. 2025; Mayden and Kuhajda 2025) with numerous impervious surfaces that result in high volumes of stormwater runoff and reduced recharge of groundwater, producing an unnatural hydrograph. This leads to streambed scouring after rain events, causing channelization and incision of stream beds and uprooting of aquatic vegetation, and exacerbated drought conditions in summer and fall further contributing to the loss of aquatic vegetation and resulting low ecological productivity. Springs within the Valley Creek watershed can mitigate low flow conditions but increased urban development and groundwater pumping put springs and their aquifers at risk (Duncan et al. 2010). The population of *E. birminghamense* in Fivemile Creek has not been observed for almost 20 years, despite a consistent documentation stretching from 1966 to 2006 and is feared extirpated (Brownstein et al. 2025) due to surface water disappearing into a sinkhole and pollution of the waterway from industrial discharge (“Black Warrior Riverkeeper - Press Releases” 2004).

We also highlight two specimen collections of single snubnose darters identified as *Etheostoma bellator* housed in the fish collection of the Auburn University Museum of Natural History fish collection (AUM). These specimens were collected in October 1971 from the Fivemile Creek system (Mayden and Kuhajda 2025), a direct tributary of Locust Fork—distinct from Fivemile Creek, which is a tributary of Valley Creek. During our October 2025 fieldwork, we attempted to collect snubnose darters from the same localities: AUM 9558 (Tarrant Spring Branch, Jefferson Co., Alabama 33.61333, −86.72639) and AUM 9624 (Fivemile Creek, Jefferson Co., Alabama 33.605, −86.73083). Despite extensive carbonate bedrock at the Fivemile Creek location, we failed to capture any snubnose darters. Other researchers have similarly failed to collect snubnose darters from Fivemile Creek over the last half century (W.M. Howell *pers. comm.* 2025), suggesting that this population, which may be distinct from all other species in the *Etheostoma chermocki* complex, is extinct. Adequate assessment of the status of Birmingham Darter in the Valley Creek system is essential for ensuring this brilliantly colored microendemic species does not suffer the same fate as this possibly distinct but undescribed species.

## Figures and Tables

**Table 1.** Full Set of Occurrences of Birmingham Darter (*Etheostoma birminghamense*) in Valley Creek watershed, Jefferson County, Alabama. New sites reported here denoted with asterisk.

Catalog	Count	Latitude	Longitude	Locality	Year	Collector
YPM ICH 033237	7	33.386487	−87.080516	Blue Creek at Johns Road (TJN19-13) Five Mile Creek at Co. Rd. 20, 0.8 mi SSE of McCalla Five Mile Creek at Freeman Ave., 1.1 mi NNW of McCalla Five Mile Creek at Freeman Avenue Five Mile Creek at Hercules Powder Plant Five Mile Creek at McCalla Five Mile Creek at old bridge along I-59/20, just NE jct I-459, 0.5 mi W of McCalla Five Mile Creek at old bridge, just downstream of I-59/20 Five Mile Creek at Old Hwy Bridge off of I-59/20 Five Mile Creek at Powder Plant in McCalla Fivemile Creek just downstream of I-59/20 Fivemile Creek, 3 mi SW of Bessemer Hospital on US Hwy 11 Fivemile Creek, 3 mi SW of Bessemer Hospital on US Hwy 11 Fivemile Creek, 3 mi SW of Bessemer Hospital on US Hwy 11 Fivemile Creek at McCalla at I-459. Fivemile Creek at McCalla. Fivemile Creek at McCalla. Fivemile Creek just S of Loiusville and Nashville Railroad line, just W of McAdory Fivemile Creek 4.7 mi. S of Bessemer, US Hwy. 11. Fivemile Creek at McCalla, Hwy. 20. Fivemile Creek at McCalla. Fivemile Creek, old Hwy. 11, 9.7 mi. S of Bessemer. Valley Creek at 19th Street in Bessemer Valley Creek at 25th Avenue just south of intersection with 19th St. Blue Creek at Johns Road Blue Creek 200 feet upstream Johns Road (Rt 36) Blue Creek at Johns Road Blue Creek at Johns Road Valley Creek along Johns Road/AL State Hwy 36 Blue Creek at Johns Road Little Blue Creek at CDX Gas Road access from Johns Road/AL State Hwy 36 Valley Creek at Williams Sr Drive, Birmingham Unnamed trib. Halls Creek near the Watercress Darter National Wildlife Refuge Seven Springs run along Cleburne Avenue in Birmingham Nabors Branch downstream of 24th St. SW at junction with Seven Springs run in Birmingham Seven Springs run along Cleburne Avenue in Birmingham Nabors Branch downstream of 24th St. SW at junction with Seven Springs run in Birmingham	2019	
UAIC 12664.01	1	33.3380556	−87.008611		1999	D.A. Neely B.R. Kuhajda, H.T.
UAIC 10670.01	4	33.3641667	−87.018611		1993	Boschung, J.R.Tomelleri B.R. Kuhajda, H.T.
UAIC 10449.01	7	33.3641667	−87.018611		1992	Boschung, W.M. Howell B.R. Kuhajda, H.T.
UAIC 10450.01	23	33.3805556	−87.025		1992	Boschung, W.M. Howell R.D. Caldwell, W.M.
UAIC 2011.02	1	33.3475	−87.022222		1966	Howell
UAIC 11898.01	1	33.3477778	−87.0225		1997	C.C. Blanco, J.C. Keiser B.R. Kuhajda, R.L. Mayden,
UAIC 11055.02	2	33.3477778	−87.0225		1994	H.T. Boschung, R.L. Mayden, J.S. Boyce,
UAIC 12246.01	2	33.3377778	−87.008611		1996	C.A. Stephens
UAIC 6481.01	2	33.3805556	−87.025		1976	A. Black, Alexander B.R. Kuhajda, H.T.
UAIC 10448.01	3	33.3477778	−87.0225		1992	Boschung, W.M. Howell R.D. Caldwell, W.M.
UAIC 1934.1	29	33.3377778	−87.008611		1966	Howell J.D. Williams, J.G.
UAIC 2504.08	4	33.3377778	−87.008611		1967	Armstrong, T.S. Jandebeur
UAIC 3041.15	11	33.3377778	−87.008611		1968	H. Harima, T.S. Jandebeur
TU 163187	9	33.3475	−87.02167		1992	R.D. Suttkus
TU 162681	42	33.3475	−87.02167		1992	R.D. Suttkus
TU 163017	49	33.3475	−87.02167		1992	R.D. Suttkus J. Khudamrongsawat, B.R.
UAIC 14638.01	6	33.3872222	−87.0225		2006	Kuhajda, J.H. Howell
TU 62670	4	33.3475	−87.02167		1969	R.D. Suttkus & Cashner
TU 140976	9	33.3475	−87.0217		1985	R.D. Suttkus
TU 140953	7	33.3475	−87.02167		1985	R.D. Suttkus
TU 188886	7	33.3377778	−87.008611		1998	K.R. Piller & J.A. Tipton B.R. Kuhajda, B.L. Fluker,
UAIC 15142.01	1	33.4194444	−86.983333		2007	M.G. Bennett
		33.4182756	−86.983316			
YPM ICH 033230	5				2019	
YPM ICH 033237	3	33.386487	−87.080516		2019	
YPM ICH 035273	6	33.386007	−87.080591		2022	
YPM ICH 037373	8	33.386487	−87.080516		2023	
YPM ICH 038290	1	33.386487	−87.080516		2023	Holotype
*YPM ICH 0XXXXX	18	33.3881839	−87.059656		2025	
*YPM ICH 0XXXXX	1	33.386487	−87.080516		2025	
*YPM ICH 0XXXXX	3	33.373968	−87.050331		2025	
*No voucher Geological Survey of Alabama	62	33.458021	−86.905023		2025	
*No voucher Geological Survey of Alabama	3	33.375004	−86.965000		2024	
*Photo voucher Tennessee Aquarium Conservation Institute	1	33.471845	−86.871249		2008	B.R. Kuhajda, D.A. Neely, S. Duncan, K. Hamm
*Photo voucher Tennessee Aquarium Conservation Institute	40^+^	33.470864	−86.875007		2020	B.R. Kuhajda, J. Drummond
*Photovoucher Tennessee Aquarium Conservation Institutye	1	33.471845	−86.871249		2025	B.R. Kuhajda, E.C. Culp, M.C. Vicente
*Photovoucher Tennessee Aquarium Conservation Institute	4	33.470864	−86.875007		2025	B.R. Kuhajda, E.C. Culp, M.C. Vicente
